# Supplementary material for: Learning curve and surgical outcome of robotic assisted colorectal surgery with ERAS program
Source: Sci Rep. 2022 Nov 29;12:20566. doi: 10.1038/s41598-022-24665-w (PMC9709162; doi:10.1038/s41598-022-24665-w)
Supplement: Supplementary file 2 — Supplementary Information 2. [file 41598_2022_24665_MOESM2_ESM.zip › RAL Raw data-Ñ[▒K/112/HS_ñw╝╨░O▒Kñσ.pdf]

正式摘要(電子簽章：000000226675739)
W83 / W83 ☐ 英文標題(注意:無英文姓名-請補病人基本檔!) ☐【讀取】憑證

臺中榮民總醫院
出院病歷摘要

轉入號： 轉出號：

[redacted]

(8)流水編號	
(9)入院日期	110/09/14 (2021) 直腸外科 W83 - 102
(10)轉科日期	
(11)出院日期	110/10/04 (2021) 住院天數計 20 日

診斷

- 入院 :Rectum Cancer, cT3N0M0, Stage II, 5cm FAV, s/p pelvic neoadjuvant radiotherapy (25Gy/5frs)2021/08/30-2021/09/03
  [PHx]
  1. Type 2 diabetes mellitus under OHA, 2021/08/19 HbA1C: 7.2
  2. Hypertension
  3. Hypertriglyceridemia
- 出院 :[A/P]
  1. Rectum Cancer, cT3(mrg-)N0M0, Stage II, 5cm FAV,
  s/p short course neoadjuvant radiotherapy (25Gy/5frs)2021/08/30-2021/09/03
  -s/p SILS robotic TME+ loop ileostomy on 2021/09/15
  pT3N0(0/15), stageIIA.
  MD, pMMR, AI-, PI-, TuDp-, DsMg:1cm-, CRM:1.5cm-, ExNd-, TRG: absent,
  r/o post-OP IAI (drain culture 9/18: E.coli, KP, 9/25: Proteus, PsA)
  s/p Flomoxef(2021/09/17-09/24), Tazocin(2021/09/24-10/4), oral Ciproxin (10/4-)
  [PHx]
  1. Type 2 diabetes mellitus under OHA, 2021/08/19 HbA1C: 7.2
  2. Hypertension
  3. Hypertriglyceridemia

主訴

- constipation for 6 m.

病史

- This 56 year old male with past history of
  1. Type 2 diabetes mellitus under OHA, 2021/08/19 HbA1C: 7.2
  2. Hypertension
  3. Hypertriglyceridemia
  He was a cook, smoking 1 PPD for 40+ years without quit, alcohol consumption 300cc whisky/高粱 for 40+ years quit for 4 months. Family history: mother had breast cancer and DM, father had hypertension.
  He was found a conlon polyp at LMD in 2014, this time, due to epigastric pain, body weight loss 5kg(76-> 71) in 3 months, and defecation habit change(tenesmus, thinner stool sometimes), he visited 賢德 LMD for UGI and LGI scope on 2021/08, which disclosed a colon polyp, 40cm to cecum moderate dysplasia, and 10cm high dysplasia. He then visited our OPD for help.
  Sigmoidscopy on 2021/08/09 disclosed a tumor at 5cm FAV s/p biopsy, pathology: adenocarcinoma, moderately differentiated. Tumor survey was done. Tumor marker on 2021/08/10: CEA: 8.7, CA199: 17.18. Abdominal CT on 2021/08/10: a 2.5cm polypoid lesion in rectum(axi#80), r/i rectal cancer may be compatible. No significant regional lymphadenopathy or intra-abdominal metastasis. Chest CT on 2021/08/12 showed no definite evidence of lung metastasis. 2021/08/19 pelvicto the subserosal tissue, compatible with colon CA. PET on 2021/08/24: no bone metastasis. Rectum Cancer, cT3N0M0, Stage II, 5cm FAV was MRI: Presence of annular wall thickening and pedunculated enhancing mass lesion about 75mm in length at the rectosigmoid colon without significant invasion suspected. Short course radiotherapy for pelvic area was suggested, he received neoadjuvant radiotherapy (25Gy/5frs) from 2021/08/30-2021/09/03. He presented with back soreness and intermittent diarrhea up to 3 times/day recently. No fever, no cough, no nausea/vomitng, no urinary symptoms. He was admitted for robotic low anterior resection and postoperative care.

體檢發現

-
  - General appearance :
    - acute ill looking , conscious clear , without cardiopulmonary distress
    - Vital signs自動帶入date 體溫(BT) 脈搏(P) 呼吸(R) 血壓(BP) 身高(BH) 體重(BW) BMI
      20210914 36.4℃ 61次/分 18次/分 113/77mmHg 171cm 68kg 23.3
    - Vital signs: B.P. 113/77 mmHg , P.R. 61 /min , R.R. 18 /min , B.T. 36.4 ℃
  - HEENT :
    - Eyes: no anemic conjunctiva, no icteric sclera, normal EOM
    - ENT: no otorrhea, no rhinorrhea, no erythematous mucosa, no injected tonsil, no deformity
  - Neck: supple, freely movable, trachea midline,no deformity, no engorged jugular vein , no palpable thyroid or lymph node enlargement
  - Chest:
    - Inspection: Grossly normal, symmetric and fully expansion, no accessory muscle used, no operation scar, no spider angiomata, no gynecomastia
    - Palpation: no palpable mass or nodule, no subcutaneous emphysema, no tenderness
    - Ausculation: clear breathing sound
  - Heart:
    - Percussion: normal heart size
    - Ausculation: regular heart beat, normal S1, S2, no gallop, no murmur
  - Abdomen:
    - Inspection: in
      - flat contour, symetrically, no scar, no stria, no dilated veins, no rashes
    - Ausculation: normoactive bowel sound, no abdominal bruits
    - palpation: soft, no muscle guarding or rigidity, no local tenderness, no rebounding pain, no palpable mass, no palpable pulsation
    - Percussion: no shifting dulllness, no hepatomegaly, no splenomagaly
  -

Peripheral pulsation :	CA	BA	RA	FA	PA	PTA	DPA
Right side :	++	++	++	++	++	++	++/+++
Left side :	++	++	++	++	++	++	++/+++

  - Genito-anus: no tenderness or palpable mass lesion or rectal shelf, normal external genitalia, normal anal tone, brown stool

CRS CONDITION

  - Colonoscope report: 2021/08/09: adenocarcinoma, moderately differentiated
  - CEA: 8.7 ng/ml
  -
    - Lung

手術日期及方法

- 手術日期及方法20210915 1. Robotic (XI) assisted TME+ Single port gelpoint.
  2. ulcerative tumor 5*5 cm ant. wall 8 cm FAV. EMR scar 25 cm FAV. Px scar 35 cm FAV wiht tatoo
  3. High ligation (+) : At IMA root. Left colic artery and IMV were
  alsoligated
  4. Indocyanine green agent was used to check the blood supply of the colon
  25mg/10cc, proxiaml: 3cc, 30 sec, distal: 2cc, 25 sec
  5. Take down from the splenic flexure : (-)
  6. Robotic endoGIA (45-4.3) X3 + EEA (29mm) were used
  7. Proximal cut end: 30 cm, distal cut end: 2 cm
  8. Hemo Vac drainage tube was inserted pelvis.
  9. protective ileostomy on RLQ

住院治療經過

- After admission, he received robotic TME+ loop ileostomy on 2021/09/15 smoothly. He tolerated the procedure well. Fever with chillness and abdominal pain were noted on 9/17 afternoon. Fever survey disclosed elevated CRP and lactate, drain culture yielded E.coli and KP. Antibiotics was shifted to Flomoxef(09/17-09/24), Tazocin(09/24-). CXR showed RLL infiltration, while KUB disclosed intestine distention and right staghorn stone. Fever subsided after change antibiotics. Foley and rod were removed on 09/22, and started to try diet with fair digestion. Turbid drainage was found on 09/25, with culture yielded Proteus and Pseudomonas, sensitive to Tazocin. H/V was removed on 09/30. We kept empirical Tazocin and switched to oral form antibiotics as Ciproxin. Due to relatively stable status, he may be discharged with CRS OPD follow up.

合併症

- nil.

一般檢查

- 累積報告：

DATE	TIME	ALB	TP	BIL.T	BIL.D	ALKP	AST	ALT	LDH	AMY	LIPASE	NH3	Ferritin	UA
1100914	1649	4.2		1.1	0.2	73	17	20	110					
1100915	1609	3.7		0.8				15						
1100917	1808	3.2		0.89				8						
1100920	0901	3.4		0.93				11						
1100923	0852	3.4		1.98				18						
1100927	0956	3.2		0.73	0.25			19						
1100930	0850	3.3		0.6	0.2			22						

DATE	TIME	WBC	RBC	HGB	HCT	MCV	PLT	BLST	NEUT	BAND	SEG	LYM	MON	EOS	BAS
1100914	1649	6540	4.70	15.0	43.1	91.7	151		74.9			14.8	6.1	3.7	0.5
1100915	1620	9990		14.8	42.0		145		89.2			6.7	3.5	0.5	0.1
1100917	1807	3140		14.5			129			4.0	79.0	15.0	2.0	0.0	0.0
1100920	0925	9830		14.6			151			7.0	86.0	2.0	4.0	1.0	0.0
1100923	0856	7020		13.7			293				78.0	6.0	13.0	1.0	0.0
1100927	0913	8160		12.8			369			1.0	82.0	7.0	5.0	3.0	0.0
1100930	0920	6580		13.5			397		80.4			10.3	5.2	3.6	0.5

DATE	TIME	NA	K	CL	BUN	CREAT	eGFR	CA	Mg	P	CRP	ESR	PCT	D-Dimer	LACTATE
1100914	1649	142	3.6	106	16	1.08		8.5							
1100915	1609	142	3.7	109	14	1.02	80.3	7.7							
1100917	1807														19.0
1100917	1808	136	4.4		11	0.94	88.24	7.9			5.727				
1100920	0901	131	3.8		24	0.98	84.09	8.8	1.5	1.6					
1100923	0852	129	3.5		13	0.89	93.98	8.2	1.7	3.1					
1100927	0956	135	3.5		10	0.84	100.46	8.5							
1100930	0850	138	3.9		10	0.94	88.24	8.7							

DATE	TIME	GLUCOSE	HbA1C	CHOL	TG	HDL	LDL	KETONE	CK	CKMB	TRO-T	PT-P	APTT-P	NT-pro BNP
1100914	1649	101										11.3	30.5	

DATE	TIME	SP-GR	PH	PROT	GLUC	KETON	BILI	UROB	OB	NITR	LEU	RBC	WBC	EPCEL
1100917	1850	1.020	5.5	Trace	Negative	Negative	Negative	0.2	3+	Negative	1+	2+	5-10	0-1
1100922	2144	1.014	6.5	Negative	250.0	Negative	Negative	0.2	3+	Negative	Negative	35-50	2-5	1-2

DATE	TIME	STOOL-OB	FAT	RBC	WBC	PARA
1100917	2016	Negative		-	-	Not Found

DATE	TIME	PH	PCO2	PO2	SO2	BEB	HCO3	TCO2	HGB	FIO2
1100917	1805	7.460	29.1	80.1	96.4	-2.2	20.2	21.1		

- 放射線報告 :檢驗項目 **:CHEST PA VIEW** 報告日期 **:1100927**

主　訴：
Result:N
Finding:
> The heart size is within normal limit
> Calcification of thoracic aorta
> Haziness opacity in right lower lung field with increased interstitial infiltration over residual lung field.
> Degenerative change of thoracic and lumbar spine with osteophytes formation.
> Old fracture of left clavicle
> on nasogastric tube, EKG leads and central line
> Presence of right staghorn stone
** Pneumoperitoneum over right subphrenic region is noted, may be post-op change, suggest follow-up and clinical correlation to r/o the possibility of hallow organ perforation
> On central line
Impression:
As above description.

檢驗項目 **:CHEST AP VIEW** 報告日期 **:1100919**

主　訴：
Result:N
Finding:
> No cardiomegaly
> Increased interstitial infiltration in bilateral lungs.
> Bilateral costophrenic angle mild blunting, pleural effusion or pleural change could be considered.
> Degenerative change of thoracic and lumbar spine with osteophytes formation.
> Radiopaque density over right upper abdomen, renal stone cannot be ruled out
> On central venous catheter
Impression:
As above description.

檢驗項目 **:ABDOMEN (KUB),SUPINE** 報告日期 **:1100920**

主　訴：
Result:N
Finding:
> Much bowel gas and fecal material is noted.
> Degenerative joint disease over thoracic and lumbar spine
> Radiopaque densities are noted over right renal region, complete staghorn stones are favored.
> S/P ostomy over RLQ area
> Surgical autosuture and drainage tube in pelvic region.
Impression:
As above description.

檢驗項目 **:CHEST AP VIEW (PORTABLE)** 報告日期 **:1100917**

主　訴：
Result:N
Finding:
> The heart size is within normal limit
> Calcification of thoracic aorta
> Mildly increased insterstitial infiltration over bilateral lung fields
> Degenerative change of thoracic and lumbar spine with osteophytes formation.
> Old fracture of left clavicle
> on CVC
> Presence of right staghorn stone
Impression:
As above description.

檢驗項目 **:CHEST AP VIEW (PORTABLE)** 報告日期 **:1100916**

主　訴：
Result:N
Finding:
> The heart size is within normal limit

- > Calcification of thoracic aorta
  > Mildly increased insterstitial infiltration over bilateral lung fields
  > Loculated pleural effusion over RUL
  > Degenerative change of thoracic and lumbar spine with osteophytes formation.
  > Old fracture of left clavicle
  > on nasogastric tube, EKG leads and central line
  > Presence of right staghorn stone
  Impression:
  As above description.

  檢驗項目 **:CHEST PA VIEW** 報告日期 **:1100917**
  主 訴：
  Result:N
  Finding:
  > The heart size is within normal limit
  > Calcification of thoracic aorta
  > Mildly increased insterstitial infiltration over bilateral lung fields
  > Loculated pleural effusion over RUL
  > Degenerative change of thoracic and lumbar spine with osteophytes formation.
  > Old fracture of left clavicle
  > on nasogastric tube, EKG leads and central line
  > Presence of right staghorn stone
  Impression:
  As above description.

特殊檢查

- PFT :nil.
- Bronchoscope :nil.
- nil.

病理報告

- 活體組織切片病理報告　手術日期：**110/08/09**　病理號：**S1024470**

診斷醫師:文美卿 報告日期:110/08/12
Pathologic diagnosis:
Rectum tumor, 5 cm from anal verge, endoscopic biopsy ---
Adenocarcinoma, moderately differentiated.
Gross description:
The specimen consists of 3 pieces of tan soft tissue, up to
0.3x0.2x0.1 cm. All for section.
Microscopic description:
Section shows colon mucosa with invasive carcinoma with glandular
structure.
Note:
The diagnosis is concurred in intradepartment consensus meeting on
2021/08/12.
#T-59300_2 #M-81403_2 2 1340 00CM0F

活體組織切片病理報告　手術日期：**110/09/15**　病理號：**S1029267**

診斷醫師:王仕卿 報告日期:110/09/23
Addendum: 2021/09/24
Pathologic diagnosis:
1. Rectum, robot low anterior resection --- a. S/P treatment with
residual adenocarcinoma, moderately differentiated. Tumor invaded to
subserosal soft tissue. The bilateral cut ends and
mesocolic/perirectal lymph nodes (0/14) are free of tumor. b.
Tubulovillous adenoma with focal high grade dysplasia.
Prognostic and predictive factor:
11. Tumor regression grading S/P CCRT: Absent, extensive residual
cancer with no evident tumor regression (poor or no response, score
3).
14. Pathological TNM Stage: ypT3N0(According to the eighth edition,
American Joint Committee on Cancer Staging Guidelines for Tumors).
15. TNM descriptors: y (Post-treatment).

Pathologic diagnosis:
1. Rectum, robot low anterior resection --- a. Adenocarcinoma,
moderately differentiated. Tumor invaded to subserosal soft tissue.
The bilateral cut ends and mesocolic/perirectal lymph nodes (0/14)
are free of tumor. b. Tubulovillous adenoma with focal high grade
dysplasia.
2. IMA root, dissection --- Unremarkable fibroadipose tissue. No
tumor seen.
3. IMV root, dissection --- Unremarkalbe fibroadipose tissue and
lymph node (0/1).
4. Rectum, cut end, distal, resection --- Chronic inflammation.
Ancillary study for diagnosis:
IHC stains for PMS2 and MSH6 are done on section L. Both above
markers are preserved.
Prognostic and predictive factor:
1. Tumor size: 3.5 cm.
2. Depth of invasion: Subserosal soft tissue.
3. Histologic grade: G2: Moderately differentiated.
4. Angiolymphatic invasion: Not identified.
5. Perineural invasion: Not identified.
6. Tumor deposits (discontinuous extramural extension): Not
identified.
7. Macroscopic tumor perforation: Not identified.
8. Surgical Margins: a) Proximal Margin: Uninvolved by invasive
carcinoma, distance of tumor from margin: 12 cm. b) Distal Margin:
Uninvolved by invasive carcinoma, distance of tumor from margin: 1
cm. c) Radial or Mesenteric Margin: Uninvolved by invasive carcinoma,
distance of tumor from margin: 1.5 cm.
9. Lymph node status: Uninvolved (0/15).
10. Extranodal involvement: Not identified.
11. Tumor regression grading S/P CCRT: No known presurgical therapy.
12. Type of polyp in which invasive carcinoma arose: Not identified.
13. Additional pathologic findings: Tubulovillous adenoma with focal
high grade dysplasia.
14. Pathological TNM Stage: pT3N0(According to the eighth edition,
American Joint Committee on Cancer Staging Guidelines for Tumors).
15. TNM descriptors: Not applicable.
Gross description:
The specimen consists of 1) a segment of rectum, 16 cm in length and
7 cm in proximal cut end circumference, 6 cm in distal circumference,
and perirectal soft tissue. A gray tan firm annular elevated and
centrally ulcerative tumor, 3.5x3 cm, is noted, 1 cm to the distal
cut end. On cross section, the tumor invades to the subserosal soft
tissue with 1.5 cm to the radial margin, and serosal retraction is
also noted. There is another polypoid tumor, 2x2x1.5 cm, 1 cm
proximal to the main tumor. The both cut ends are free of tumor

- grossly. 2) IMA root, a piece of tan yellow soft tissue, 2x2x1 cm. 3) IMV root, a piece of tan yellow soft tissue, 2.5x2x1 cm. 4) distal cut end, a piece of tan mucosa-covered soft tissue, 2.5x2x1 cm. Representative part for section: A) specimen 2 B) specimen 3 C) specimen 4 D) proximal cut end E) radial margin F-G) polyp H-M) main tumor N-R) lymph nodes.
  Note:
  1. The interpretation of MMR panel and recommended studies are as below: (1) No loss of nuclear expression of MMR proteins: low probability of microsatellite instability-high (MSI-H) . (2) Loss of nuclear expression of MLH1 and PMS2: testing for methylation of the MLH1 promoter and/or mutation of BRAF is indicated (the presence of a BRAF V600E mutation and/or MLH1 methylation suggests that the tumor is sporadic and germline evaluation is probably not indicated; absence of both MLH1 methylation and of BRAF V600E mutation suggests the possibility of Lynch syndrome, and sequencing and/or large deletion/duplication testing of germline MLH1 may be indicated). (3) Loss of nuclear expression of MSH2 and MSH6: high probability of Lynch syndrome (sequencing and/or large deletion/duplication testing of germline MSH2 may be indicated, and, if negative, sequencing and/or large deletion/duplication testing of germline MSH6 may be indicated). (4) Loss of nuclear expression of MSH6 only: high probability of Lynch syndrome (sequencing and/or large deletion/duplication testing of germline MSH6 may be indicated). (5) Loss of nuclear expression of PMS2 only: high probability of Lynch syndrome (sequencing and/or large deletion/duplication testing of germline PMS2 may be indicated). (6) Please note: There are exceptions to the above IHC interpretations. These results should not be considered in isolation, and clinical correlation with genetic counseling is recommended to assess the need for germline testing.
  2. This case has been peer reviewed by two doctors.
  #T-59600_2 #M-81403_2 2 1105 S0000F
  #T-59600_2 #M-82630_2 0 1105 000000
  #T-C4560_2 #M-09183_2 0 1105 000000

其它

- **檢驗項目 :Bacterial Culture(Anaerobic) 檢體:Drainage(hemovac) 報告日期 :1100929**

附註 :
陰性結果 : No growth for anaerobic culture.
陽性結果 : ISOLATED ORGANISM: COLONY COUNT:( )/ml
(1)
(2)
(3)
菌落量定義 : P:Pure H:Heavy M:Moderate S:Slight moderate F:Few E:Enrich
SUSCEPTIBILITY TEST: S:Susceptible I:Intermediate R:Resistant D: Susceptible Dose Dependent (SDD) NS: Nonsusceptible

	(1)	(2)	(3)	(4)
Ampicillin				
Cefazolin				
Clindamycin				
Clarithromycin				
Gentamicin				
Amikacin				
Chloramphenicol				
Ampicillin/Sulbactam				
Penicillin				
Gentamicin(120ug)				
Trimethoprim+Sulfamethoxazole				
Vancomycin				
Oxacillin				
Cefpirome				
Ciprofloxacin				
Minocycline				
Carbapen (IPM)				
Cefoperazone/Sulbactam				
Cefotaxime				
Ceftazidime				
Cefepime				
Ceftriaxone				
Flomoxef				
Levofloxacin				
Teicoplanin				
Piperacillin/Tazobactam				
Cefmetazole				
Aztreonam				
Tigecycline				
Amoxicillin/Clavulanic acid				
Cefoxitin				
Metronidazole				
Carbapen (MPM)				
Erythromycin				
Moxifloxacin				
Linezolid				
Ertapenem				
Colistin				
Daptomycin				

注意事項 :

檢驗項目 :Bacterial Culture(Ordinary) 檢體:Drainage(hemovac) 報告日期 :1100928

附註 :
陰性結果 :
陽性結果 : ISOLATED ORGANISM: COLONY COUNT:( )/ml
(1) Proteus mirabilis H
(2) Pseudomonas aeruginosa H
(3)
菌落量定義 : P:Pure H:Heavy M:Moderate S:Slight moderate F:Few E:Enrich
SUSCEPTIBILITY TEST: S:Susceptible I:Intermediate R:Resistant D: Susceptible Dose Dependent (SDD) NS: Nonsusceptible

	(1)	(2)	(3)	(4)
Ampicillin				
Cefazolin	S=4			
Clindamycin				
Clarithromycin				
Gentamicin	S=1	S		
Amikacin	S=2	S		
Chloramphenicol				
Ampicillin/Sulbactam	S=2			
Penicillin				
Gentamicin(120ug)				
Trimethoprim+Sulfamethoxazole	S=20			
Vancomycin				
Oxacillin				
Cefpirome				

• Ciprofloxacin	S=0.25	S
Minocycline		
Carbapen (IPM)	I=2	S
Cefoperazone/Sulbactam	S=8	S
Cefotaxime		
Ceftazidime	S=0.12	S
Cefepime	S=0.12	S
Ceftriaxone	S=0.25	
Flomoxef	S=2	
Levofloxacin		
Teicoplanin		
Piperacillin/Tazobactam	S=4	S
Cefmetazole		
Aztreonam		
Tigecycline		
Amoxicillin/Clavulanic acid		
Cefoxitin	S=4	
Metronidazole		
Carbapen (MPM)		
Erythromycin		
Moxifloxacin		
Linezolid		
Ertapenem	S=0.12	
Colistin		
Daptomycin		

注意事項： For Enterobacteriaceae :

1.對於腸內菌屬的敏感性試驗，cefepime若採用CLSI M100-24的判讀標準，當試驗結果為敏感性時，其建議治療劑量為1gm Q12H；若以最低抑制濃度（MIC）的方法，結果為敏感劑量依賴性（susceptible-dose dependent；SDD）時，當MIC為 4 ug/mL時則建議劑量為1gm/Q8H或2gm/Q12H，若是MIC為8 ug/mL時或採用紙錠擴散法結果為SDD，則建議治療劑量為2gm/Q8H。

2.Cefazolin predict results for the oral cephalosporins agents (cefaclor, cephalexin) when used for therapy of uncomplicated UTIs due to E. coli, K. pneumoniae, and P. mirabilis.

3.Cefoperazone/Sulbactam目前並無國際性的判讀標準，現行引用文獻的判讀規範，僅供臨床參考。

For Pseudomonas aeruginosa:

1.Cefoperazone/Sulbactam目前並無國際性的判讀標準，現行引用文獻的判讀規範，僅供臨床參考。

檢驗項目 :**Blood Culture** 檢體:**Blood** 報告日期 :**1100925**

附註 ：

陰性結果： No bacterial growth for 7 days.

陽性結果： ISOLATED ORGANISM: COLONY COUNT:( )/ml

(1)

(2)

(3)

菌落量定義： P:Pure H:Heavy M:Moderate S:Slight moderate F:Few E:Enrich

SUSCEPTIBILITY TEST: S:Susceptible I:Intermediate R:Resistant D: Susceptible Dose Dependent (SDD) NS: Nonsusceptible

	(1)	(2)	(3)	(4)
Ampicillin				
Cefazolin				
Clindamycin				
Clarithromycin				
Gentamicin				
Amikacin				
Chloramphenicol				
Ampicillin/Sulbactam				
Penicillin				
Gentamicin(120ug)				
Trimethoprim+Sulfamethoxazole				
Vancomycin				
Oxacillin				
Cefpirome				
Ciprofloxacin				
Minocycline				
Carbapen (IPM)				
Cefoperazone/Sulbactam				
Cefotaxime				
Ceftazidime				
Cefepime				
Ceftriaxone				
Flomoxef				
Levofloxacin				
Teicoplanin				
Piperacillin/Tazobactam				
Cefmetazole				
Aztreonam				
Tigecycline				
Amoxicillin/Clavulanic acid				
Cefoxitin				
Metronidazole				
Carbapen (MPM)				
Erythromycin				
Moxifloxacin				
Linezolid				
Ertapenem				
Colistin				
Daptomycin				

注意事項：

檢驗項目 :**Bacterial Culture(Ordinary)** 檢體:**Sputum** 報告日期 :**1100920**

附註 ： I n a d e q u a t e s p e c i m e n

陰性結果： Normal mixed flora presented.

陽性結果： ISOLATED ORGANISM: COLONY COUNT:( )/ml

(1)

(2)

(3)

菌落量定義： P:Pure H:Heavy M:Moderate S:Slight moderate F:Few E:Enrich

SUSCEPTIBILITY TEST: S:Susceptible I:Intermediate R:Resistant D: Susceptible Dose Dependent (SDD) NS: Nonsusceptible

	(1)	(2)	(3)	(4)
Ampicillin				
Cefazolin				
Clindamycin				
Clarithromycin				
Gentamicin				
Amikacin				
Chloramphenicol				
Ampicillin/Sulbactam				
Penicillin				
Gentamicin(120ug)				
Trimethoprim+Sulfamethoxazole				

- Vancomycin
  Oxacillin
  Cefpirome
  Ciprofloxacin
  Minocycline
  Carbapen (IPM)
  Cefoperazone/Sulbactam
  Cefotaxime
  Ceftazidime
  Cefepime
  Ceftriaxone
  Flomoxef
  Levofloxacin
  Teicoplanin
  Piperacillin/Tazobactam
  Cefmetazole
  Aztreonam
  Tigecycline
  Amoxicillin/Clavulanic acid
  Cefoxitin
  Metronidazole
  Carbapen (MPM)
  Erythromycin
  Moxifloxacin
  Linezolid
  Ertapenem
  Colistin
  Daptomycin
  注意事項：

檢驗項目 :Gram Stain 檢體:Sputum 報告日期 :1100918
1) PMN >25 /LPF
2) Squamous epithelial cell >25 /LPF
3) Moderate Gram postive cocci in chain
4) Few Gram postive cocci in group
5) Few Gram negative cocci
6) Few Gram negative bacilli
7)
8)
備註:
@ FEW: 1 - 5, MODERATE: 6 - 30, HEAVY: > 30

檢驗項目 :Bacterial Culture(Ordinary) 檢體:Urine-voided midstream 報告日期 :1100920
附註 :
陰性結果： No growth for ordinary culture.
陽性結果： ISOLATED ORGANISM: COLONY COUNT:( )/ml
(1)
(2)
(3)
菌落量定義： P:Pure H:Heavy M:Moderate S:Slight moderate F:Few E:Enrich
SUSCEPTIBILITY TEST: S:Susceptible I:Intermediate R:Resistant D: Susceptible Dose Dependent (SDD) NS: Nonsusceptible

	(1)	(2)	(3)	(4)
Ampicillin				
Cefazolin				
Clindamycin				
Clarithromycin				
Gentamicin				
Amikacin				
Chloramphenicol				
Ampicillin/Sulbactam				
Penicillin				
Gentamicin(120ug)				
Trimethoprim+Sulfamethoxazole				
Vancomycin				
Oxacillin				
Cefpirome				
Ciprofloxacin				
Minocycline				
Carbapen (IPM)				
Cefoperazone/Sulbactam				
Cefotaxime				
Ceftazidime				
Cefepime				
Ceftriaxone				
Flomoxef				
Levofloxacin				
Teicoplanin				
Piperacillin/Tazobactam				
Cefmetazole				
Aztreonam				
Tigecycline				
Amoxicillin/Clavulanic acid				
Cefoxitin				
Metronidazole				
Carbapen (MPM)				
Erythromycin				
Moxifloxacin				
Linezolid				
Ertapenem				
Colistin				
Daptomycin				

注意事項：

檢驗項目 :Bacterial Culture(Anaerobic) 檢體:Drainage(hemovac) 報告日期 :1100923
附註 :
陰性結果： No growth for anaerobic culture.
陽性結果： ISOLATED ORGANISM: COLONY COUNT:( )/ml
(1)
(2)
(3)
菌落量定義： P:Pure H:Heavy M:Moderate S:Slight moderate F:Few E:Enrich
SUSCEPTIBILITY TEST: S:Susceptible I:Intermediate R:Resistant D: Susceptible Dose Dependent (SDD) NS: Nonsusceptible

	(1)	(2)	(3)	(4)
Ampicillin				
Cefazolin				
Clindamycin				
Clarithromycin				
Gentamicin				
Amikacin				
Chloramphenicol				
Ampicillin/Sulbactam				

- Penicillin
  Gentamicin(120ug)
  Trimethoprim+Sulfamethoxazole
  Vancomycin
  Oxacillin
  Cefpirome
  Ciprofloxacin
  Minocycline
  Carbapen (IPM)
  Cefoperazone/Sulbactam
  Cefotaxime
  Ceftazidime
  Cefepime
  Ceftriaxone
  Flomoxef
  Levofloxacin
  Teicoplanin
  Piperacillin/Tazobactam
  Cefmetazole
  Aztreonam
  Tigecycline
  Amoxicillin/Clavulanic acid
  Cefoxitin
  Metronidazole
  Carbapen (MPM)
  Erythromycin
  Moxifloxacin
  Linezolid
  Ertapenem
  Colistin
  Daptomycin
  注意事項：

檢驗項目 :Bacterial Culture(Ordinary) 檢體:Drainage(hemovac) 報告日期 :1100922
附註 ：
陰性結果：
陽性結果： ISOLATED ORGANISM: COLONY COUNT:( )/ml
(1) Escherichia coli F
(2) Klebsiella pneumoniae F
(3)
菌落量定義： P:Pure H:Heavy M:Moderate S:Slight moderate F:Few E:Enrich
SUSCEPTIBILITY TEST: S:Susceptible I:Intermediate R:Resistant D: Susceptible Dose Dependent (SDD) NS: Nonsusceptible

	(1)	(2)	(3)	(4)
Ampicillin				
Cefazolin	R>=64	S=4		
Clindamycin				
Clarithromycin				
Gentamicin	S=1	S=1		
Amikacin	S=2	S=2		
Chloramphenicol				
Ampicillin/Sulbactam	R>=32	S=2		
Penicillin				
Gentamicin(120ug)				
Trimethoprim+Sulfamethoxazole	S=20	S=20		
Vancomycin				
Oxacillin				
Cefpirome				
Ciprofloxacin	I=0.5	S=0.25		
Minocycline				
Carbapen (IPM)	S=0.25	S=0.25		
Cefoperazone/Sulbactam	S=8	S=8		
Cefotaxime				
Ceftazidime	R=32	S=0.25		
Cefepime	S=0.12	S=0.12		
Ceftriaxone	R=32	S=0.25		
Flomoxef	I=16	S=2		
Levofloxacin				
Teicoplanin				
Piperacillin/Tazobactam	S=4	S=4		
Cefmetazole				
Aztreonam				
Tigecycline				
Amoxicillin/Clavulanic acid				
Cefoxitin	R>=64	S=4		
Metronidazole				
Carbapen (MPM)				
Erythromycin				
Moxifloxacin				
Linezolid				
Ertapenem	S=0.12	S=0.12		
Colistin				
Daptomycin				

注意事項： For Enterobacteriaceae :

1.對於腸內菌屬的敏感性試驗，cefepime若採用CLSI M100-24的判讀標準，當試驗結果為敏感性時，其建議治療劑量為1gm Q12H；若以最低抑制濃度（MIC）的方法，結果為敏感劑量依賴性（susceptible-dose dependent；SDD）時，當MIC為 4 ug/mL時則建議劑量為1gm/Q8H或2gm/Q12H，若是MIC為8 ug/mL時或採用紙錠擴散法結果為SDD，則建議治療劑量為2gm/Q8H。

2.Cefazolin predict results for the oral cephalosporins agents (cefaclor, cephalexin) when used for therapy of uncomplicated UTIs due to E. coli, K. pneumoniae, and P. mirabilis.

3.Cefoperazone/Sulbactam目前並無國際性的判讀標準，現行引用文獻的判讀規範，僅供臨床參考。

檢驗項目 :Stool Culture 檢體:Stool 報告日期 :1100920
附註 ：
陰性結果： Neg. for Salmonella, Shigella and Vibrio
陽性結果： ISOLATED ORGANISM: COLONY COUNT:( )/ml
(1)
(2)
(3)
菌落量定義： P:Pure H:Heavy M:Moderate S:Slight moderate F:Few E:Enrich
SUSCEPTIBILITY TEST: S:Susceptible I:Intermediate R:Resistant D: Susceptible Dose Dependent (SDD) NS: Nonsusceptible
(1) (2) (3) (4)
Ampicillin
Cefazolin
Clindamycin
Clarithromycin
Gentamicin
Amikacin
Chloramphenicol
Ampicillin/Sulbactam
Penicillin

- Gentamicin(120ug)
  Trimethoprim+Sulfamethoxazole
  Vancomycin
  Oxacillin
  Cefpirome
  Ciprofloxacin
  Minocycline
  Carbapen (IPM)
  Cefoperazone/Sulbactam
  Cefotaxime
  Ceftazidime
  Cefepime
  Ceftriaxone
  Flomoxef
  Levofloxacin
  Teicoplanin
  Piperacillin/Tazobactam
  Cefmetazole
  Aztreonam
  Tigecycline
  Amoxicillin/Clavulanic acid
  Cefoxitin
  Metronidazole
  Carbapen (MPM)
  Erythromycin
  Moxifloxacin
  Linezolid
  Ertapenem
  Colistin
  Daptomycin
  注意事項：

檢驗項目 :**Blood Culture** 檢體:**Blood** 報告日期 :**1100923**
附註 ：
陰性結果： No bacterial growth for 7 days.
陽性結果： ISOLATED ORGANISM: COLONY COUNT:( )/ml
(1)
(2)
(3)
菌落量定義： P:Pure H:Heavy M:Moderate S:Slight moderate F:Few E:Enrich
SUSCEPTIBILITY TEST: S:Susceptible I:Intermediate R:Resistant D: Susceptible Dose Dependent (SDD) NS: Nonsusceptible
(1) (2) (3) (4)
Ampicillin
Cefazolin
Clindamycin
Clarithromycin
Gentamicin
Amikacin
Chloramphenicol
Ampicillin/Sulbactam
Penicillin
Gentamicin(120ug)
Trimethoprim+Sulfamethoxazole
Vancomycin
Oxacillin
Cefpirome
Ciprofloxacin
Minocycline
Carbapen (IPM)
Cefoperazone/Sulbactam
Cefotaxime
Ceftazidime
Cefepime
Ceftriaxone
Flomoxef
Levofloxacin
Teicoplanin
Piperacillin/Tazobactam
Cefmetazole
Aztreonam
Tigecycline
Amoxicillin/Clavulanic acid
Cefoxitin
Metronidazole
Carbapen (MPM)
Erythromycin
Moxifloxacin
Linezolid
Ertapenem
Colistin
Daptomycin
注意事項：

檢驗項目 :**Blood Culture** 檢體:**Blood** 報告日期 :**1100923**
附註 ：
陰性結果： No bacterial growth for 7 days.
陽性結果： ISOLATED ORGANISM: COLONY COUNT:( )/ml
(1)
(2)
(3)
菌落量定義： P:Pure H:Heavy M:Moderate S:Slight moderate F:Few E:Enrich
SUSCEPTIBILITY TEST: S:Susceptible I:Intermediate R:Resistant D: Susceptible Dose Dependent (SDD) NS: Nonsusceptible
(1) (2) (3) (4)
Ampicillin
Cefazolin
Clindamycin
Clarithromycin
Gentamicin
Amikacin
Chloramphenicol
Ampicillin/Sulbactam
Penicillin
Gentamicin(120ug)
Trimethoprim+Sulfamethoxazole
Vancomycin
Oxacillin
Cefpirome
Ciprofloxacin
Minocycline
Carbapen (IPM)
Cefoperazone/Sulbactam

- Cefotaxime
  Ceftazidime
  Cefepime
  Ceftriaxone
  Flomoxef
  Levofloxacin
  Teicoplanin
  Piperacillin/Tazobactam
  Cefmetazole
  Aztreonam
  Tigecycline
  Amoxicillin/Clavulanic acid
  Cefoxitin
  Metronidazole
  Carbapen (MPM)
  Erythromycin
  Moxifloxacin
  Linezolid
  Ertapenem
  Colistin
  Daptomycin
  注意事項：

出院時情況

- 3.改門診治療

出院指示

藥名	劑量	劑量單位	用法	頻次	數量	數量單位	現況	天數	單位
Dobecon tab 100mg	1	TAB	PO	TIDAC	27	TAB	TH	9	天
Loperamide cap 2mg	2	CAP	PO	BID	36	CAP	TH	9	天
Anxiedin tab 0.5mg	1	TAB	PO	QN	9	TAB	TH	9	天
Ultracet tab	1	TAB	PO	Q8HPRN	27	TAB	TH	9	天
Voren-K tab 25mg	1	TAB	PO	BIDPRN	18	TAB	TH	9	天
(□)Takepron OD tab 30mg	1	TAB	PO	QDAC	9	TAB	TH	9	天
(錠)Ciproxin tab 250mg	2	TAB	PO	Q12H	28	TAB	TH	7	天

出院計劃

- 門診日期
  - date on 2021/10/12

主治醫師 2222H 林俊余 / 住院醫師 9727A 林順鴻

正式摘要(電子簽章：000000226675739)

W83 / W83

doctorRank:B
